# Antibacterial Properties of a Novel Zirconium Phosphate-Glycinediphosphonate Loaded with Either Zinc or Silver

**DOI:** 10.3390/ma12193184

**Published:** 2019-09-28

**Authors:** Davide Campoccia, Stefano Ravaioli, Riccardo Vivani, Anna Donnadio, Eleonora Vischini, Alessandro Russo, Livia Visai, Carla Renata Arciola, Lucio Montanaro, Morena Nocchetti

**Affiliations:** 1 Laboratorio di Patologia delle Infezioni Associate all’Impianto, IRCCS Istituto Ortopedico Rizzoli, via di Barbiano 1/10, 40136 Bologna, Italy; stefano.ravaioli@ior.it (S.R.); lucio.montanaro@unibo.it (L.M.); 2 Department of Pharmaceutical Sciences, University of Perugia, Via del Liceo, 1, 06123 Perugia, Italy; anna.donnadio@unipg.it (A.D.); eleonoravischini@yahoo.it (E.V.); morena.nocchetti@unipg.it (M.N.); 3 Clinica Ortopedica e Traumatologica II, IRCCS Rizzoli Orthopaedic Institute, 40136 Bologna, Italy; alessandro.russo@ior.it; 4 Molecular Medicine Department (DMM), Center for Health Technologies (CHT), UdR INSTM, University of Pavia, Pavia, Italy and Department of Occupational Medicine, Toxicology and Environmental Risks, Istituti Clinici Scientifici (ICS) Maugeri, IRCCS, 27100 Pavia, Italy; livia.visai@unipv.it; 5 Department of Experimental, Diagnostic and Specialty Medicine, University of Bologna, via San Giacomo 14, 40126 Bologna, Italy

**Keywords:** implant infections, silver nanoparticles, zirconium phosphonates, antibacterial activity, *S. aureus*, *S. epidermidis*, *E. faecalis*, *E. coli*, *P. aeruginosa*

## Abstract

A novel compound consisting of a zirconium phosphate-glycinediphosphonate (ZPGly) has recently been introduced. This 2D-structured material forming nanosheets was exfoliated under appropriate conditions, producing colloidal aqueous dispersions (ZPGly-e) which were then loaded with zinc (Zn/ZPGly) or silver ions. Silver ions were subsequently reduced to produce metallic silver nanoparticles on exfoliated ZPGly nanosheets (Ag@ZPGly). In the search for new anti-infective materials, the present study investigated the properties of colloidal dispersions of ZPGly-e, Zn/ZPGly, and Ag@ZPGly. Ag@ZPGly was found to be a bactericidal material and was assayed to define its minimal inhibitory concentration (MIC) and minimal bactericidal concentration (MBC) on the five most prevalent pathogens of orthopaedic implant infections, namely: *Staphylococcus aureus* ATCC25923, *Staphylococcus epidermidis* RP62A, *Enterococcus faecalis* ATCC29212, *Escherichia coli* ATCC51739, and *Pseudomonas aeruginosa* ATCC27853. MIC and MBC were in the range of 125–250 μg/mL and 125–1000 μg/mL, respectively, with *E. coli* being the most sensitive species. Even colloidal suspensions of exfoliated ZPGly nanosheets and Zn/ZPGly exhibited some intrinsic antibacterial properties, but only at greater concentrations. Unexpectedly, Zn/ZPGly was less active than ZPGly-e.

## 1. Introduction

Infections are a main cause of the failure of, and one of the most serious complications associated with the use of, indwelling prosthetic devices, particularly in orthopedics [1]. The worryingly increasing antibiotic resistance among clinical bacterial strains suggests that, in future, there will be diminished possibilities for a medical cure of infections, and higher mortality for patients bearing prosthetic devices [1,2].

The development of anti-infective biomaterials that are capable of contrasting bacterial colonization and biofilm formation on implant surfaces appears to be one of the strategies with the greatest potential to prevent implant infections. However, the development of new bulk materials which are intrinsically bactericidal and which do not compromise either the mechanical or the biological properties of the implant has proved to be very challenging. Conversely, the solution of thin antibacterial coatings applied onto prosthetic surfaces appears more feasible. Thus, over the years, research efforts have been directed towards producing material surfaces which are more and more resistant to bacterial adhesion and which are functionalized with additional beneficial properties, such as anti-corrosion and anti-wear properties [3,4,5]. Biomaterial coatings are being designed to selectively modify the superficial properties of the implant, influencing host protein adsorption and promoting desirable interactions with host cells while hindering bacterial colonization [6,7,8]. Thin coatings can be used as delivery systems for the release of bioactive ions and molecules that are capable of exerting a controlled bactericidal action [9]. The efforts made to develop successful coatings which are capable of warding off implant infections have multiplied over the years [10,11,12]. In view of the decreasing efficacy of antibiotic substances against populations of bacteria which are increasingly drug-resistant, attention has been directed toward alternative antibacterial bioactive principles, including disinfectant molecules [13,14], metal ions and nanoparticles [15], nanocomposites [16], phytochemicals [17], and antimicrobial peptides [18].

Zirconium phosphate nanostructured materials, especially in form of nanoplatelets, have been reportedly described as convenient delivery systems, with superior drug delivery functionality with respect to other inorganic layered materials [19,20], high loading capacity, and a lack of cytotoxicity [21]. Known as excellent inorganic cation exchange materials, zirconium phosphates exhibit thermal stability and are capable of incorporating a variety of compounds, with large molecules such as proteins and enzymes among them [22]. The envisioned uses of zirconium phosphates include the release of chemotherapeutic drugs in cancer therapy, where hexagonal nanoplatelets have been explored as a platform for drug delivery to treat cancer [23].

Recently, a novel compound consisting of zirconium phosphate-phosphonate, namely zirconium phosphate-glycinediphosphonate (ZPGly), based on *N*,*N*-bis-(phosphonomethyl)glycine and with formula Zr_2_(PO_4_)[(HO_3_PCH_2_)NHCH_2_COOH]-[(O_3_PCH_2_)_2_NHCH_2_-COOH]⋅H_2_O was introduced [24]. It can be easily synthesized in mild conditions, and its crystal structure was first solved from X-ray powder diffraction data [24]. This compound was found to exhibit a layered structure and to crystallize in the monoclinic C2/c space group. Each layer has been found to be about 15 Å thick and to consist of a double plane of ZrO_6_ octahedra connected by tetradentate PO_4_ groups lying in an intermediate plane. The glycine moieties were reported to be placed on the external part of the layers and connected to the zirconium atoms through the PO_3_C groups [24]. Due to the presence of glycine and P-OH groups which are exposed on the surface of layers, this compound showed excellent ion exchange properties (ion exchange capacity, IEC = 3.67 mmol/g), with a high affinity for transition metals [25]. The use of metals such as silver and zinc (but also copper and gallium) in anti-infective coatings has always been the object of much attention. However, over the last few years, interest for these metals has multiplied. These metals could offer a real possible alternative to the use of anti-infective biomaterials based on conventional antibiotics [26] which are losing their efficacy due to the increase in antibiotic resistance among clinical isolates [27]. Moreover, recent studies have shown that nanoparticles of metals such as silver are not only effective against both planktonic and sessile bacteria within biofilms [28], but they have also been found to act synergistically with conventional antibiotics [29]. In view of all these considerations, the present study aims at investigating the microbiological properties of ZPGly and of two related compounds obtained by loading ZPGly with zinc ions or silver nanoparticles. The biological properties of these novel materials, only recently developed and characterized, appear to be still largely unexplored.

## 2. Materials and Methods

### 2.1. Test Materials

All chemicals for the preparation of the test materials were purchased from Sigma-Aldrich (Milan, Italy). ZPGly powders were synthesized as earlier described [24]. Briefly, 2.37 g of newly-prepared *N,N*-bis-(phosphonomethyl)glycine was solubilized in 93 mL of deionized water and a volume of 6 mL of 1 M phosphoric acid was added to the solution. This solution was mixed in a Teflon bottle with 20.4 mL of a 2.9 M hydrofluoric acid solution containing 6 mmol of zirconium oxychloride octahydrate and heated in an oven and maintained at a temperature of 90 °C for 3 days. The solid was then filtered under vacuum, washed 3 times with deionized water, and dried at 60 °C for 24 h. The procedure for the preparation of colloidal dispersions of ZPGly decorated with silver nanoparticles (Ag@ZPGly) or exchanged with zinc ions (Zn/ZPGly) is reported in ref. [30] and is briefly recalled here.

#### 2.1.1. Ag@ZPGly

A suspension of 300 mg of ZPGly (0.367 mmol) in 29 mL of deionized water was exfoliated with 11 mL of 0.1 M methylamine (1.1 mmol corresponding to 100% of ZPGly IEC). The dispersion was kept under vigorous magnetic stirring at room temperature for 24 h. Then, 20 mL of 1 M hydrochloric acid was added in order to remove methylamine and convert the acidic groups on the surface of layers in protonate form. The obtained gel was washed twice with deionized water, recovered by centrifugation (13,000 rpm, 10 min) and redispersed in 15 mL of deionized water, yielding a colloidal dispersion of exfoliated ZPGly nanosheets (ZPGly-e) with a concentration of 19.6 mg of dry solid/mL. Next, 2.1 mL of 0.06 M AgCH_3_COO aqueous solution were added dropwise under vigorous stirring to 15 mL of this dispersion and left under magnetic agitation for 1 day. The solid was recovered by centrifugation (15,000 rpm for 10 min) and washed twice with water. The sample was equilibrated for 12 h at room temperature in 70 mL of ethanol in order to reduce Ag and produce silver nanoparticles (Ag NPs). Finally, the dispersed solid was recovered by centrifugation, washed twice with water, and dispersed again in 15 mL of de-ionized water. This final dispersion contained 20.2 mg of dry solid/mL. The dry solid contained 3.52 % w/w of Ag.

#### 2.1.2. Zn/ZPGly

A volume of 2.2 mL of 0.25 M Zn(CH_3_COO)_2_ aqueous solution was added dropwise under stirring to 15 mL of ZPGly-e (prepared as above described). The mixture was left under stirring for 24 h. After washing with deionized water, the recovered gel was dispersed in 15 mL of deionized water. This final dispersion contained 19.1 mg of solid/mL. The dry solid contained 13.1 % w/w of Zn.

### 2.2. Bacterial Strains

For the study of the antibacterial properties of the test materials, five different reference bacterial strains were selected as being representative of the most prevalent etiologic agents causing implant related infections. The strains used were, respectively: *Staphylococcus aureus* ATCC^®^25923, a clinical isolate regularly used as reference strain in antibiotic susceptibility testing; *Staphylococcus epidermidis* ATCC^®^35984, a biofilm-forming strain isolated from an intravascular catheter-associated sepsis, alternatively known as RP62A; *Enterococcus faecalis* ATCC^®^29212, a CLSI quality control and susceptibility testing clinical reference strain originally isolated from a urine sample; *Escherichia coli* ATCC^®^51739, a reference strain used for quality control of strain typing; and *Pseudomonas aeruginosa* ATCC^®^27853, a reference strain for antibiotic susceptibility testing isolated from a hospital blood specimen. *S. aureus*, *S. epidermidis*, *E. faecalis* and the two Gram-negative species, respectively *P. aeruginosa* and *E. coli*, are known to be the principal opportunistic pathogens causing biomaterial associated infections, in particular those related to orthopedic implants [31]. Bacterial isolates were thawed from frozen stocks of the strain library of the Research Unit on Implant Infections and plated on Tryptical Soy Agar (MEUS S.r.l., Piove di Sacco, Italy).

### 2.3. MIC (Minimal Inhibitory Concentration) and MBC (Minimal Bactericidal Concentration) Tests

For the minimal inhibitory concentration (MIC) test, a few colonies of each bacterial strain, taken from the TSA plates, were resuspended in TSB (Biolife Italiana srl, Milan, Italy) and were cultured at 37 °C. After about 3 h of incubation, the bacterial suspensions were initially diluted to a concentration of approximately 10^8^ CFU/mL estimated by optical density reading at 625 nm, using a Hewlett Packard G1103A spectrophotometer (Waldbronn, Germany). The bacterial suspension was further diluted to a concentration of 1:100 in TSB. Serial 1:2 dilutions of the powder suspensions of the test materials were prepared in TSB starting from stock solutions of approximately 20 mg/mL of the material in deionized water. Each microtiter plate was prepared by adding a volume of 100 µL of inoculum to 100 µL of diluted material suspension. Each dilution was assayed in triplicate wells. The final concentration of bacteria was of approximately 5 × 10^5^ CFU/mL, and the range of concentrations of the test materials was in the range between 2000 µg/mL and 0.98 µg/mL. The microplates included triplicate control wells with sterile TSB and TSB inoculated with the bacterium. At time 0 and after one day of incubation at 37 °C, the optical density of the plates was read at a wavelength of 600 nm using a Modulus II multifunction plate reader (Turner BioSystems, Sunnyvale, CA, USA). The measure of the optical density at time = 0 was subtracted from the final reading at 1 day in order to account for the turbidity caused by the particles of the test materials in suspension at the highest concentrations. MIC was estimated from the curves obtained by plotting the results of up to 5 (generally 3) independent experiments conducted at different times. Conversely, in order to assess the minimal bactericidal concentration (MBC), the entire volume of bacterial suspension was removed from each well at the end of the treatment and was plated on TSA. After incubation at 37 °C for 1 day, the agar plates were examined for bacterial growth and CFU were eventually counted. The MBC was calculated as the concentration causing at least a 3 LOG reduction with respect to the initial inoculum (i.e., a count ≤ 100 CFU per plate).

## 3. Results

### Evaluation of the Antibacterial Activity of the Different ZPGly-Based Compounds

The antibacterial activity of the test materials in form of colloidal dispersion was assessed by determining the MIC and MBC by the serial dilution technique. For the MIC, the plates were optically read by a plate reader. The subtraction of the optical reading of the plates at time 0, i.e., before bacterial growth, accounted for some level of the cloudiness observed at the two highest concentrations of the material dispersions. The tests were performed in a range of concentration of 1–2000 µg/mL. The use of greater concentrations of materials in the tests was avoided because of the increased optical cloudiness, but also in order to avoid an excess of broth dilution when starting from aqueous suspensions of about 20 mg/mL.

The results of the MIC and MBC tests conducted with the three test materials on *S. epidermidis* RP62A and *E. coli* ATCC^®^51739 are reported in Table 1 and Table 2. Ag@ZPGly exhibited a powerful antimicrobial activity against these two bacterial species, with a MIC value as low as 125 µg/mL corresponding to a total silver concentration of 4.4 µg/mL. For the *E. coli* ATCC^®^51739 strain the MBC value was found to be just slightly higher than the MIC value. In contrast, Ag@ZPGly was found to be bactericidal on *S. epidermidis* at a minimal concentration of 1000 µg/mL, showing a larger gap between MIC and MBC.

**Figure 1:**
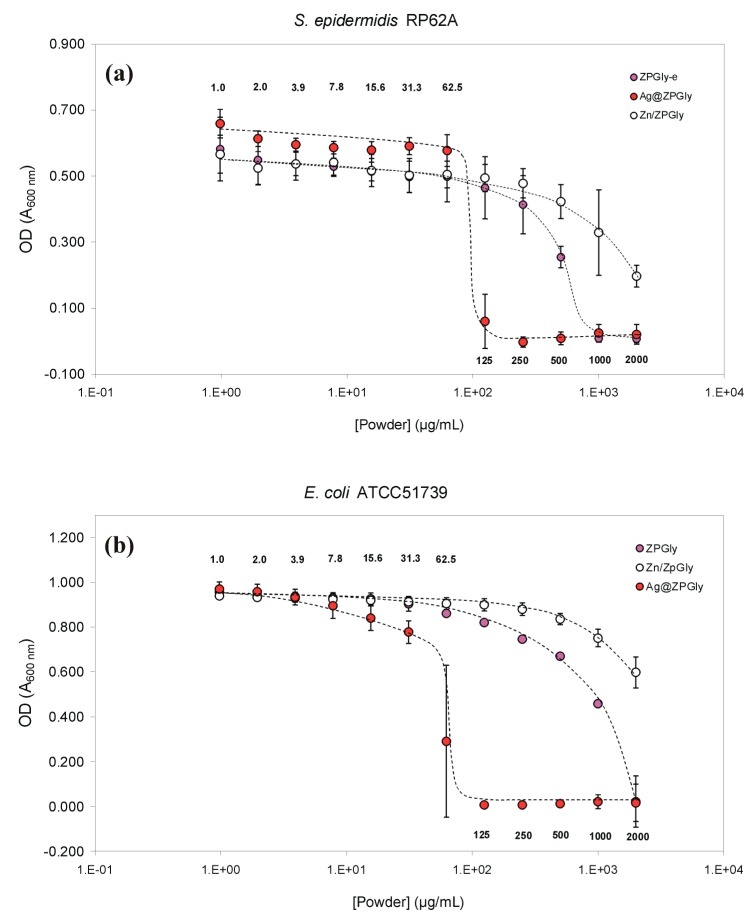
Inhibition curves for the three test materials added to bacterial suspensions of: (**a**) the *S. epidermidis* strain RP62A and (**b**) the *E. coli* ATCC^®^51739. Data reported in the plot correspond to the mean OD values of the triplicate wells of the independent experiments performed ± S.D.

**Figure 2:**
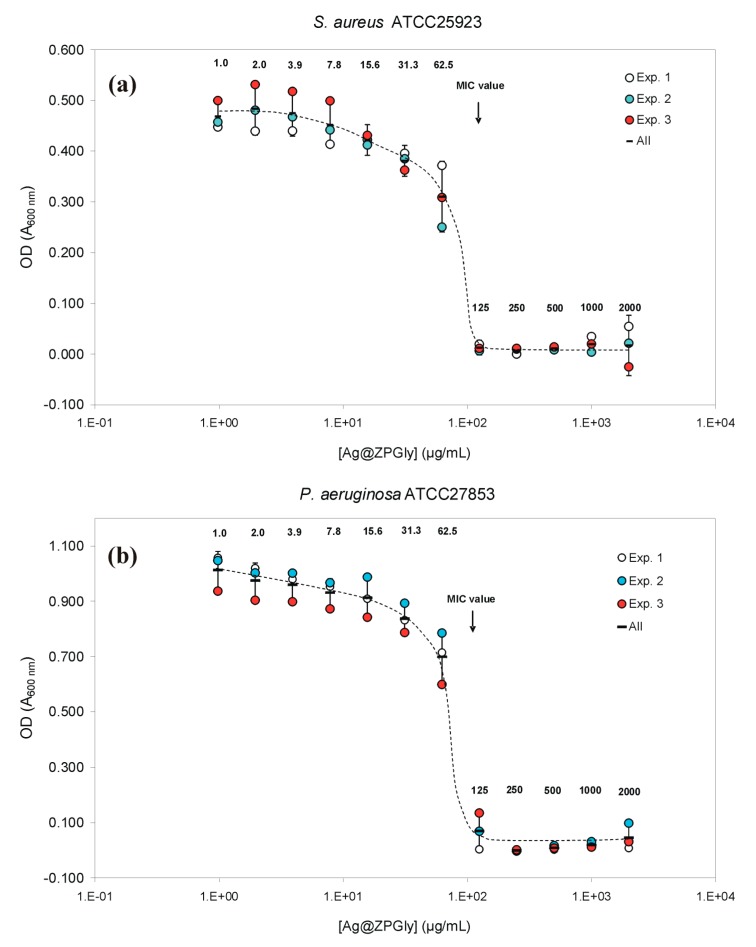
Bacterial inhibition curves by Ag@ZPGly: (**a**) *S. aureus* ATCC^®^25923 and (**b**) *P. aeruginosa* ATCC^®^27853. Data reported in the plot correspond to the mean OD values found for each single experiment and the mean of the 3 independent experiments ± S.D.

The other two test materials showed inferior antimicrobial properties. Indeed, the MIC of ZPGly powders with *S. epidermidis* RP62A and *E. coli* ATCC^®^51739 was respectively of 1000 µg/mL and 2000 µg/mL, and the MIC of Zn/ZPGly even exceeded 2000 µg/mL (Figure 1 and Figure 2), suggesting a lower antibacterial activity of these two materials on these bacterial strains. On the front of MBC, these last two compounds did not show microbicidal activity in the range of concentration tested. In view of the lower antimicrobial activity observed for ZPGly-e and Zn/ZPGly, attention was therefore primarily focused on Ag@ZPGly, the test material exhibiting the greatest bactericidal potential against *S. epidermidis* and *E. coli*.

The Ag@ZPGly confirmed the same MIC value of 125 µg/mL as observed for *S. epidermidis* and *E. coli*, even when tested on *S. aureus* ATCC^®^25923 and *P. aeruginosa* ATCC^®^27853 (Table 3). However, on these two further pathogens, the MBC was slightly lower than that observed in the case of the coagulase-negative species, corresponding to 500 µg/mL for the *S. aureus* strain and to 1000 µg/mL for *P. aeruginosa*. The MIC value was found to double when the treatment with Ag@ZPGly dispersion was performed on *E. faecalis* (Table 3), with this species showing the lowest susceptibility to the anti-infective material. Notwithstanding, the MBC did not differ with respect to *S. epidermidis* and *P. aeruginosa*.

The different bacterial inhibition curves found for the three test materials when they were added to bacterial suspensions of the *S. epidermidis* strain RP62A are shown in Figure 1a. For Ag@ZPGly, the minimal inhibitory concentration was reached with concentrations as low as 125 µg/mL. Conversely, the ZPGly-e became inhibitory only at concentrations approaching 1 mg/mL and, for Zn/ZPGly, not even a concentration of 2 mg/mL gave complete inhibition (about 65% inhibition). Thus, the presence of Zn^2+^ not only was not found to boost the bacterial growth inhibition of ZPGly, but it would appear that the presence of this metal in the nanostructured material could even somehow attenuate the inhibitory effects observed for the metal-free ZPGly-e.

The inhibition curves of *S. aureus* by Ag@ZPGly are shown in Figure 2a. The MIC was reached with concentrations as low as 125 µg/mL, corresponding to the same MIC found also for *S. epidermidis*.

**Figure 3:**
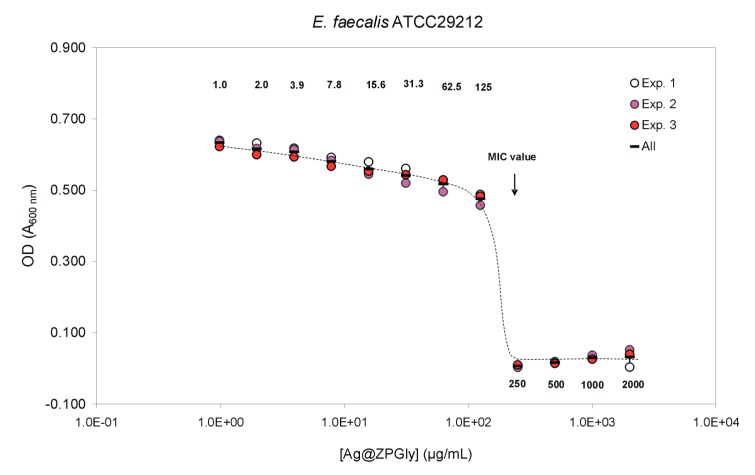
*E. faecalis* inhibition curves by Ag@ZPGly. Data reported in the plot correspond to the mean OD values found for each single experiment and the mean of the 3 experiments ± S.D.

Inhibition curves of Ag@ZPGly on *P. aeruginosa* are shown in Figure 2b. The MIC was reached with concentrations as low as 125 µg/mL. *E. faecalis* inhibition curves by Ag@ZPGly are shown in Figure 3. The MIC was reached with concentrations of 250 µg/mL, suggesting a lower susceptibility of this pathogen to silver ions released from the material.

With the *E. coli* strain ATCC^®^51739 (Figure 1b), the MIC was reached with concentrations as low as 125 µg/mL. However, some degree of inhibition was observed already at 62.5 μg/mL. This would suggest a greater susceptibility with respect to the other bacterial species here investigated.

## 4. Discussion

Bacteria are prone to colonizing artificial surfaces forming biofilms, i.e., populations of bacteria stuck in bio-polymeric matrices [32,33]. Biofilms are decisive in determining the irreducibility of implant infections. When a biofilm is copious and mature, some bacteria leave the nestled consortium and migrate to a new site where they anchor and settle. Thus, the cycle of the biofilm starts over and the infection perpetuates and spreads [33,34]. Therefore, the *primum movens* of the biofilm cycle and infection establishment is the bacterial adhesion and colonization on the biomaterial surface [32,35]. Here, new ZPGly-based materials were investigated to ascertain their antibacterial activity and assess their potential for the future production of coatings which are capable of counteracting bacterial adhesion/colonization, and thus, of preventing implant infections.

In this explorative study, ZPGly-based materials were tested in the form of stable colloidal dispersions.

All materials were found to exhibit antibacterial properties, even in the absence of metal loading, as intrinsic antimicrobial properties were observed also for ZPGly-e. Nonetheless, silver-loaded ZPGly exhibited the greatest activity, causing bacterial inhibition already at concentrations as low as 125–250 µg/mL, i.e., 1 Log lower than those of ZPGly-e. Conversely, the loading with Zn^2+^ ions was not found to confer improved antibacterial performance.

As far as the MBC test is concerned, the *E. coli* strain was found to be the most sensitive to the Ag-doped compound. Ruparelia et al. [36] investigated strain specificity in the antimicrobial activity of silver nanoparticles and reported the existence of marked variations of MIC/MBC values depending on the *E. coli* strain. Conversely, with *S. aureus*, the changes observed on different strains were just negligible. In detail, the MBC for Ag NPs was found to span from 60 to 220 µg/mL in four different *E. coli* strains, while three distinct strains of *S. aureus* showed exactly the same MIC value of 160 µg/mL. Thus, the lower MBC observed in the present study could likely depend on the specific *E. coli* strain investigated.

Interestingly, in our experiment, performed on five different bacterial pathogens, the MIC and MBC values for Ag@ZPGly corresponded to loaded amounts of Ag NPs in the range of 4.4–8.8 µg/mL and 8.8–35.2 µg/mL, respectively. The observed MIC values appear similar to those reported for silver nanoparticles in a recently published study [37], where tests were performed on the same reference strains of *S. aureus* and *P. aeruginosa* as those used in the present study, and on an additional, non-well identified, methicillin-resistant *S. aureus* (MRSA). In detail, Jadhav et al. (2016) [37] found that MICs obtained by visual inspection of tube cultures for silver nanoparticles were in the range of 0.99–7.93 µg/mL. Nonetheless, some differences emerged from the comparison of the bactericidal activity. The MBC was 31.75 µg/mL for the MRSA strain, 7.93 µg/mL for *P. aeruginosa* ATCC^®^27853, and greater than the highest silver nanoparticle concentration tested for *S. aureus* ATCC^®^25923 [37]. Higher MIC and MBC values, respectively of 120 and 160 µg/mL, were reported for silver nanoparticles tested on *S. aureus* ATCC25923 by Ruparelia et al. (2008) [36].

For its intrinsic bactericidal/bacteriostatic activities, silver is currently being closely studied for its anti-infective properties in biomedical applications. There are diverse known mechanisms by which silver can exert its antimicrobial action. These mechanisms have recently been reviewed by Wahab et al. (2018) [38]. Ag^+^ ions can interact with the SH-containing groups of proteins of bacterial cell wall or plasma membrane [39] and disrupt bacterial membranes, introducing holes by which cytoplasmic content may flow out, thus causing bacterial cell death. Inside the microbial cell, Ag^+^ ions, besides inhibiting many enzymes, lead to the formation of reactive oxygen species (ROS), which are toxic to bacterial cells [40]. Unlike antibiotic resistance, bacterial resistance to silver is not commonly observed, presumably due to the multiple antimicrobial mechanisms underlying the bactericidal activity of silver, while antibiotics have specific and singular mechanisms of action. Metallic silver in the form of silver nanoparticles has made a remarkable comeback as a potential antimicrobial agent, and Ag-nanoparticles have been designed and introduced as a new generation of antimicrobials, especially when used as coatings. Nanosilver proves to be more active than silver bulk materials, probably because it can reach bacteria in closest proximity with a higher surface/mass ratio, achieving higher local concentrations of Ag^+^ and, consistently, higher bactericidal effects [41]. It is therefore expected that nanotechnology can open new ways to fight and prevent infectious diseases by using the atomic scale customization of materials.

Metal phosphonates have been shown to be materials suitable for several purposes: as heterogeneous catalysts for the synthesis of fine chemicals, as solid sorbents for gas separation, notably CO_2_ capture, as materials for electrochemical devices, such as fuel cells and rechargeable batteries, and as matrices for drug delivery [25], but descriptions of applications of Ag^+^ or Zn^2+^ metal phosphonates as antimicrobial are, until now, scarce. The antibacterial properties and biocompatibility of nanocoatings of silver-containing phosphonate monolayers formed on titanium have earlier been demonstrated by Tîlmaciu et al. (2015) [42]. Coating strategies with subnanomolar amounts of silver exposed at the outer surface were found to be suitable for preventing bacterial adhesion and biofilm formation. Interestingly, they can be applied to metallic or ceramic medical devices without compromising their biocompatibility.

Finally, it has emerged from different studies that Ag^+^ can act synergistically with other antibacterial substances, including Zn^2+^ [43] and conventional antibiotic substances [44]. In combined administration with silver, even ineffective antibiotics could be resurrected and reacquire their full effectiveness against MRSA [44]. Thus, the future development of multifunctional zirconium phosphate/phosphonate materials doped with combinations of silver and zinc or silver and antibiotics could represent a valid strategy to further boost the antibacterial action of these compounds.

Our explorative findings certainly motivate us to extend our work with these materials, not in form of colloidal suspensions, but in the more definitive form of coatings applied onto solid material surfaces. With coated materials, further biological characterization will need to be performed, investigating relevant aspects such as bacterial adhesion, surface contact killing, inhibition of biofilm formation, and all the interactions possible for bacteria and host cells on a modified solid surface.

## 5. Conclusions

In summary, the antibacterial properties of three different zirconium phosphate/phosphonate (ZPGly) compounds as colloidal nanoparticles with zinc (Zn/ZPGly) or with silver (Ag@ZPGly) were assayed for their antimicrobial activity. The Ag@ZPGly nanoparticles showed consistent anti-microbial properties, especially against *E. coli*, but more generally against the entire panel of reference strains, including the five most frequent causative agents of implant associated infections. Nonetheless, even ZPGly-e exhibited appreciable activity against *S. epidermidis* and *E. coli*, but only at 1 Log greater concentrations. Intriguingly, Zn/ZPGly was found to be less active than ZPGly-e, with its MIC slightly exceeding the range of concentrations explored. It remains to be elucidated if the lack of activity of the zinc-loaded material is also partly associated with the reduced release of the metal entrapped within the nanosheet structure.

## Figures and Tables

**Table 1:** MIC and MBC values for zirconium phosphate/phosphonate dispersions tested on *S. epidermidis* RP62A.

**Test Material**	**MIC (µg/mL)**	**Active Metal (µg/mL)**
ZPGly-e	1000	-
Zn/ZPGly	>2000	>261 (Zn)
Ag@ZPGly	125	4.4 (Ag)
**Test Material**	**MBC (µg/mL)**	**Active Metal (µg/mL)**
ZPGly-e	>2000	-
Zn/ZPGly	>2000	>261 (Zn)
Ag@ZPGly	1000	35.2 (Ag)

**Table 2:** MIC and MBC values for zirconium phosphate/phosphonate dispersions tested on *E. coli* ATCC^®^51739.

**Test Material**	**MIC (µg/mL)**	**Active Metal (µg/mL)**
ZPGly-e	2000	-
Zn/ZPGly	>2000	>261 (Zn)
Ag@ZPGly	125	4.4 (Ag)
**Test Material**	**MBC (µg/mL)**	**Active Metal (µg/mL)**
ZPGly-e	>2000	-
Zn/ZPGly	>2000	>261 (Zn)
Ag@ZPGly	250	8.8 (Ag)

**Table 3:** MIC and MBC values for Ag@ZPGly.

**Pathogen**	**MIC (µg/mL)**	**Silver (µg/mL)**
*S. aureus* ATCC25923	125	4.4
*E. coli* ATCC51739	125	4.4
*P. aeruginosa* ATCC27853	125	4.4
*E. faecalis* ATCC29212	250	8.8
**Pathogen**	**MBC (µg/mL)**	**Silver (µg/mL)**
*S. aureus* ATCC25923	500	17.6
*E. coli* ATCC51739	250	8.8
*P. aeruginosa* ATCC27853	1000	35.2
*E. faecalis* ATCC29212	1000	35.2
